# Risks Associated with High-Dose *Lactobacillus rhamnosus* in an *Escherichia coli* Model of Piglet Diarrhoea: Intestinal Microbiota and Immune Imbalances

**DOI:** 10.1371/journal.pone.0040666

**Published:** 2012-07-27

**Authors:** Xiao-Qiong Li, Yao-Hong Zhu, Hong-Fu Zhang, Yuan Yue, Zheng-Xing Cai, Qing-Ping Lu, Lu Zhang, Xiao-Gang Weng, Fan-Jian Zhang, Dong Zhou, Jin-Cai Yang, Jiu-Feng Wang

**Affiliations:** 1 College of Veterinary Medicine, China Agricultural University, Beijing, China; 2 State Key Laboratory of Animal Nutrition, Institute of Animal Sciences, Chinese Academy of Agricultural Sciences, Beijing, China; 3 Department of Animal Science, Aarhus University, Tjele, Denmark; Louisiana State University and A & M College, United States of America

## Abstract

Probiotic could be a promising alternative to antibiotics for the prevention of enteric infections; however, further information on the dose effects is required. In this study, weanling piglets were orally administered low- or high-dose *Lactobacillus rhamnosus* ACTT 7469 (10^10^ CFU/d or 10^12^ CFU/d) for 1 week before F4 (K88)-positive *Escherichia coli* challenge. The compositions of faecal and gastrointestinal microbiota were recorded; gene expression in the intestines was assessed by real-time PCR; serum tumour necrosis factor-α (TNF-α) concentrations and intestinal Toll-like receptor 4 (TLR4) were detected by ELISA and immunohistochemistry, respectively. Unexpectedly, high-dose administration increased the incidence of diarrhoea before F4^+^ETEC challenge, despite the fact that both doses ameliorated F4^+^ETEC-induced diarrhoea with increased *Lactobacillus* and *Bifidobacterium* counts accompanied by reduced coliform shedding in faeces. Interestingly, *L. rhamnosus* administration reduced *Lactobacillus* and *Bifidobacterium* counts in the colonic contents, and the high-dose piglets also had lower *Lactobacillius* and *Bacteroides* counts in the ileal contents. An increase in the concentration of serum TNF-α induced by F4^+^ETEC was observed, but the increase was delayed by *L. rhamnosus*. In piglets exposed to F4^+^ETEC, jejunal TLR4 expression increased at the mRNA and protein levels, while jejunal interleukin (IL)-8 and ileal porcine β-defensins 2 (pBD2) mRNA expression increased; however, these increases were attenuated by administration of *L. rhamnosus*. Notably, expression of jejunal TLR2, ileal TLR9, Nod-like receptor NOD1 and TNF-α mRNA was upregulated in the low-dose piglets after F4^+^ETEC challenge, but not in the high-dose piglets. These findings indicate that pretreatment with a low dose of *L. rhamnosus* might be more effective than a high dose at ameliorating diarrhoea. There is a risk that high-dose *L. rhamnosus* pretreatment may negate the preventative effects, thus decreasing the prophylactic benefits against potential enteric pathogens. Our data suggest a safe threshold for preventative use of probiotics in clinical practice.

## Introduction

Postweaning diarrhoea (PWD) mainly occurs within the first week after weaning and affects pigs across the globe, causing great economic loss to the swine industry due to reduced growth performance and considerable morbidity and mortality [1], [2]. The disease is largely caused by Enterotoxigenic *Escherichia coli* (ETEC), which are characterized by colonization to the gut epithelium through adhesion to specific receptors on the brush border membrane. Following colonization, the excretion of heat-stable enterotoxins, heat-labile enterotoxins, and *E. coli* enterotoxin 1 induces intestinal inflammatory responses and causes diarrhoea [1], [3]. The most prevalent ETEC strain implicated in PWD expresses the F4 (K88)^+^ fimbriae [1], [4]. The contribution of nontherapeutical antibiotics to the development of antibiotic resistance in both animal and human medicine leads the European Union to terminate the use of antibiotic growth promoters in food animals [5]. With a recent large outbreak of a novel multidrug-resistant *E. coli* strain O104:H4 in Germany [6], the development of alternatives to conventional antibiotics is urgently needed.

Probiotics are defined as ‘live microorganisms, which when administered in adequate amounts confer a health benefit to the host’; therefore, they represent a promising alternative to antibiotics for controlling enteric infections. As a major component of the gut microbiota, certain *Lactobacillus* species that function as probiotics may protect the host against enteric pathogens. *Lactobacillus rhamnosus* GG (LGG) appears efficacious for preventing acute infectious diarrhoea in children [7], and ETEC-induced diarrhoea in piglets [8]. In contrast, the negative effect on health of F4^+^ETEC challenged pigs after supplementation with LGG has been reported [9]. Although several possible mechanisms involved in the effects of *L. rhamnosus* have been investigated, including competitive exclusion or inhibition of pathogens, enhancement of epithelial barrier function, and modulation of both local and systemic host immune responses [10], little is known regarding the action of probiotics on the intestinal mucosa *in vivo*.

The mucosal immune system detects microorganisms by discriminating between mutualism and pathogenicity using a sophisticated system of receptors, including membrane-bound Toll-like receptors (TLRs) and cytoplasmic Nod-like receptors (NLRs). In general, signalling via TLRs or NLRs leads to the production of pro-inflammatory cytokines, chemokines and antimicrobial peptides, thereby contributing to host defense and inflammation [11], [12]. At mucosal sites, F4^+^ETEC adhere to mucosa via specific F4 receptors, thereby activating TLR4- or TLR5- mediated inflammatory responses upon recognition of LPS or flagellin by epithelial cells [13], [14]. In the case of probiotic bacteria, *in vitro* studies have shown that the anti-inflammatory function of *L. rhamnosus*, at least in part, is mediated by TLRs [15]–[17]. Of the NLRs, NOD1 and NOD2 respond to the synthetic peptidoglycan components meso-diaminopimelic acid and muramyl dipeptide, respectively [18]. The expression of both of these receptors can be enhanced by interaction with *Lactobacilli* and TLR ligands in gut-associated lymphoid tissues (GALT) [19], [20]. NOD1 and NOD2 are also involved in the recognition of intestinal commensal bacteria, thereby contributing to gut homeostasis [21], [22]. However, the relationship between probiotics, TLRs and NLRs and their role in protecting the host gut against enteric pathogens remains unknown.

The actions of probiotics are generally considered in a strain-dependent and dose-dependent manner. So far, few dose-comparison studies have been undertaken and definite recommendations on appropriate probiotics dosing regimens are unavailable [23]. It has been proposed that the beneficial effects of probiotics on ameliorating acute infectious diarrhoea are correlated with dose (greater for doses >10^10^–10^11^ colony-forming unites (CFU)/d) [24]. Controversially, a previous study on the treatment of infants with acute watery diarrhoea reported that using *L casei* at a high dose (6 to 8×10^11^ CFU/d) failed to show a positive effect [25], whereas a recent study found that both doses of LGG (10^10^ and 10^12^ CFU/d, respectively) had the efficacy in controlling acute watery diarrhoea [26]. The mechanisms underlying dose effects of probiotics are unclear, and it is unknown if increased doses (based on an effective dose >10^10^ CFU/d) reduce the incidence of diarrhoea or otherwise increase the risk of adverse immune stimulation.

Therefore, in this study, a low- and high-dose pretreatment of *L. rhamnosus* ATCC 7469 (10^10^ and 10^12^ CFU/d, respectively) was used to elucidate dose effects of *L. rhamnosus* on the gut microbiota and mucosal immune responses in F4^+^ETEC challenged pig models. Our findings suggest that pretreatment with a low dose of *L. rhamnosus* is more effective at ameliorating F4^+^ETEC-induced diarrhoea than is pretreatment with a high dose. Pretreatment with high doses may negate the preventative effects of this probiotic, thus limiting its effectiveness as a preventative in F4^+^ETEC infection.

## Results

### Low-dose *L. rhamnosus* Consumption was More Effective at Ameliorating F4^+^ETEC-induced Diarrhoea

**Figure 1:**
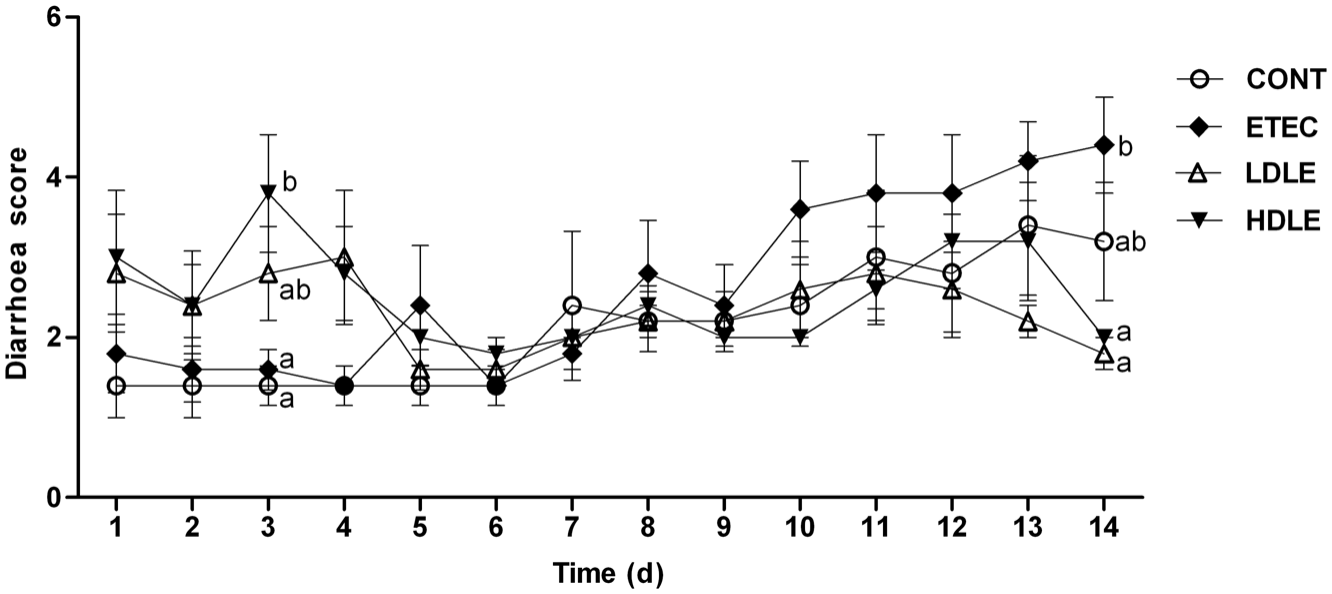
Dose effects of *L. rhamnosus* on diarrhoea scores of piglets following F4^+^ETEC challenge. Piglets received sterile physiological saline orally (CONT); received sterile physiological saline orally followed by F4^+^ETEC challenge (ETEC); were pretreated with low-dose 10^9^ CFU/ml *L. rhamnosus* solution for 1 week followed by F4^+^ETEC challenge (LDLE); or were pretreated with high-dose 10^11^ CFU/ml *L. rhamnosus* solution for 1 week followed by F4^+^ETEC challenge (HDLE). Data are presented as means ± SEM (n = 5 per group). Within the same time, mean values with different superscript letters (^a, b^) are significantly different (P<0.05).

Dose effects of *L. rhamnosus* on diarrhoea scores of piglets following the challenge were monitored each day during the 2-week experimental trial. Average daily gain and feed intake were unaffected in the first 2 weeks following the challenge (data not shown). Based on our observation, the administration of low-dose *L. rhamnosus* achieved better clinical outcomes with a reduced incidence of diarrhoea and lower diarrhoea scores, whereas high-dose *L. rhamnosus* was associated with disturbance of the microbial ecosystem, resulting in triggering of diarrhoea. Specifically, during week 1 (prior to the F4^+^ETEC challenge), only one pig in the control group (CONT) had experienced naturally acquired diarrhoea by day 7; however, pigs in the high-dose probiotic group (HDLE) had significantly higher diarrhoea scores (*P* = 0.001) than did those in group CONT, due to the incidence of severe diarrhoea (faecal scores ≥5) in HDLE pigs of 60% by day 3. By comparison, there were no notable differences in diarrhoea scores among groups ETEC (ETEC challenged pigs without probiotic treatment), LDLE (low-dose probiotic group) and CONT before the F4^+^ETEC challenge (Figure 1).

During week 2, following F4^+^ETEC challenge, 60% of ETEC pigs exhibited severe diarrhoea from day 10 to day 14, whereas only 10% of LDLE pigs and 40% of HDLE pigs had diarrhoea. ETEC pigs had higher average diarrhoea scores (3.57) than did CONT (2.14), LDLE (2.34) and HDLE (2.49) pigs. At day 14, diarrhoea scores were significantly higher in ETEC than either in LDLE (*P*<0.001) or in HDLE (*P* = 0.001) pigs.

### *Lactobacillus rhamnosus* Consumption Shaped the Composition of Faecal Microbiota During F4^+^ETEC Infection

**Figure 2:**
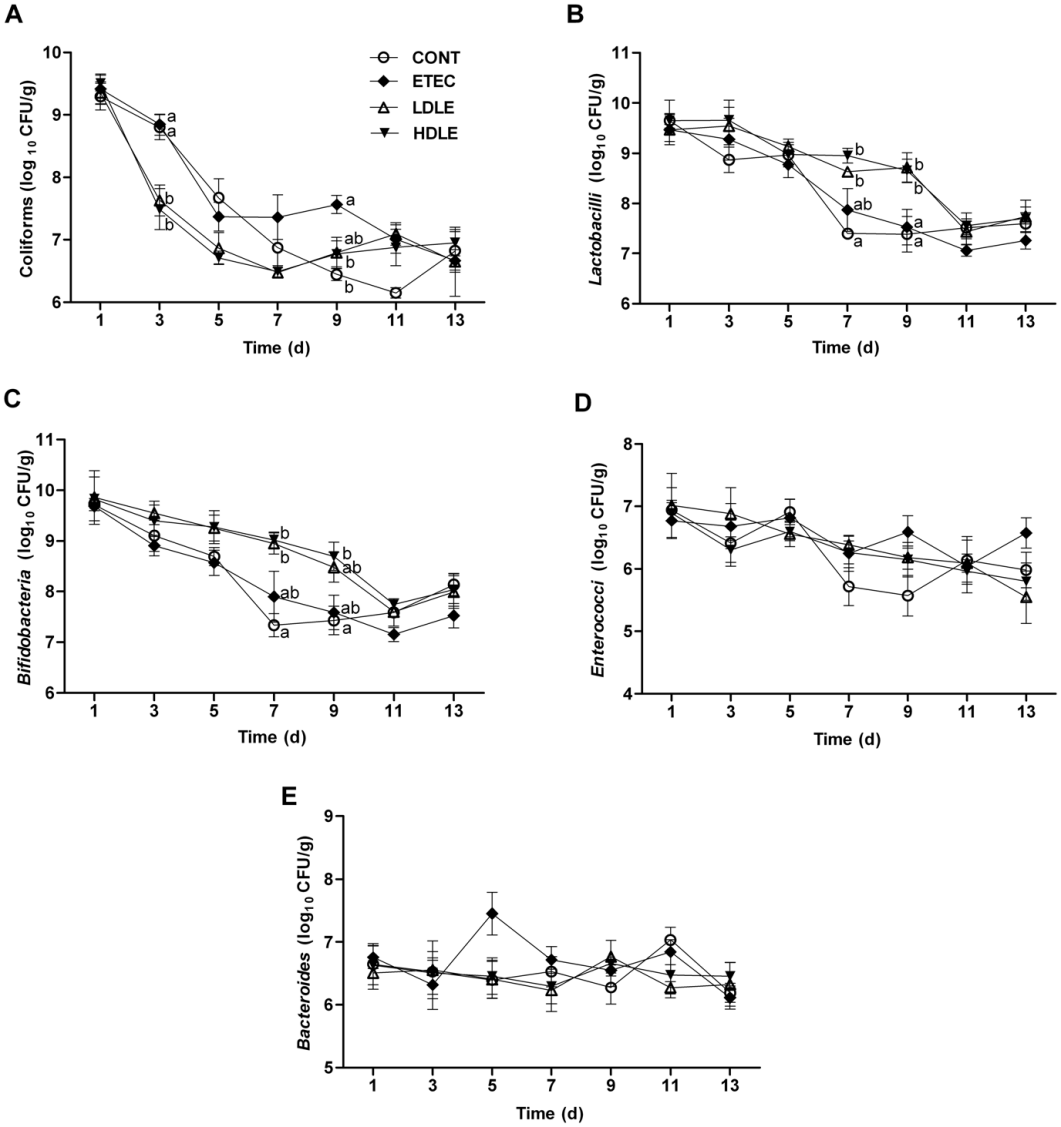
*Lactobacillus rhamnosus* consumption shaped the composition of faecal microbiota during F4^+^ETEC infection. (A) Coliforms, (B) *Lactobacilli*, (C) *Bifidobateria*, (D) *Enterococci* and (E) *Bacteroides*. Fresh faecal samples from all the piglets were collected at days 1, 3, 5, 7, 9, 11 and 13 after weaning. Results are presented as log_10_ CFU/g faeces, and all counts were performed in triplicate. Data are presented as means ± SEM (n = 5 per group). Within the same time, mean values with different superscript letters (^a, b^) are significantly different (P<0.05).

To validate whether ingested exogenous probiotics were able to colonize intestines and exert protective activity through competitive exclusion of F4^+^ETEC invasion, we monitored faecal microbial populations every two days. Both doses of *L. rhamnosus* reduced faecal coliform shedding and expanded commensal bacterial populations. At day 3, prior to F4^+^ETEC challenge, pigs pretreated with *L. rhamnosus* (group LDLE or HDLE) had a lower coliform shedding (*P* = 0.018 or *P* = 0.003, respectively) than did CONT pigs (Figure 2A). In contrast, faecal *Lactobacillus* and *Bifidobacterium* counts were notably higher in LDLE and HDLE than in CONT at day 7 (Figures 2B and 2C).

Following F4^+^ETEC challenge at day 9, faecal coliform counts were higher in ETEC than either in CONT (*P* = 0.009) or in LDLE (*P* = 0.032). In contrast, faecal *Lactobacillus* counts remained significantly higher in LDLE and HDLE than either in CONT or in ETEC. Similarly, faecal *Bifidobacterium* counts were higher (*P* = 0.019) in HDLE than in CONT. The faecal *Enterococcus* and *Bacteroides* populations were unaffected by *L. rhamnosus* administration with or without F4^+^ETEC challenge (Figures 2D and 2E).

### *Lactobacillus rhamnosus* Consumption Shaped the Composition of Intestinal Microbiota During F4^+^ETEC Infection

**Figure 3:**
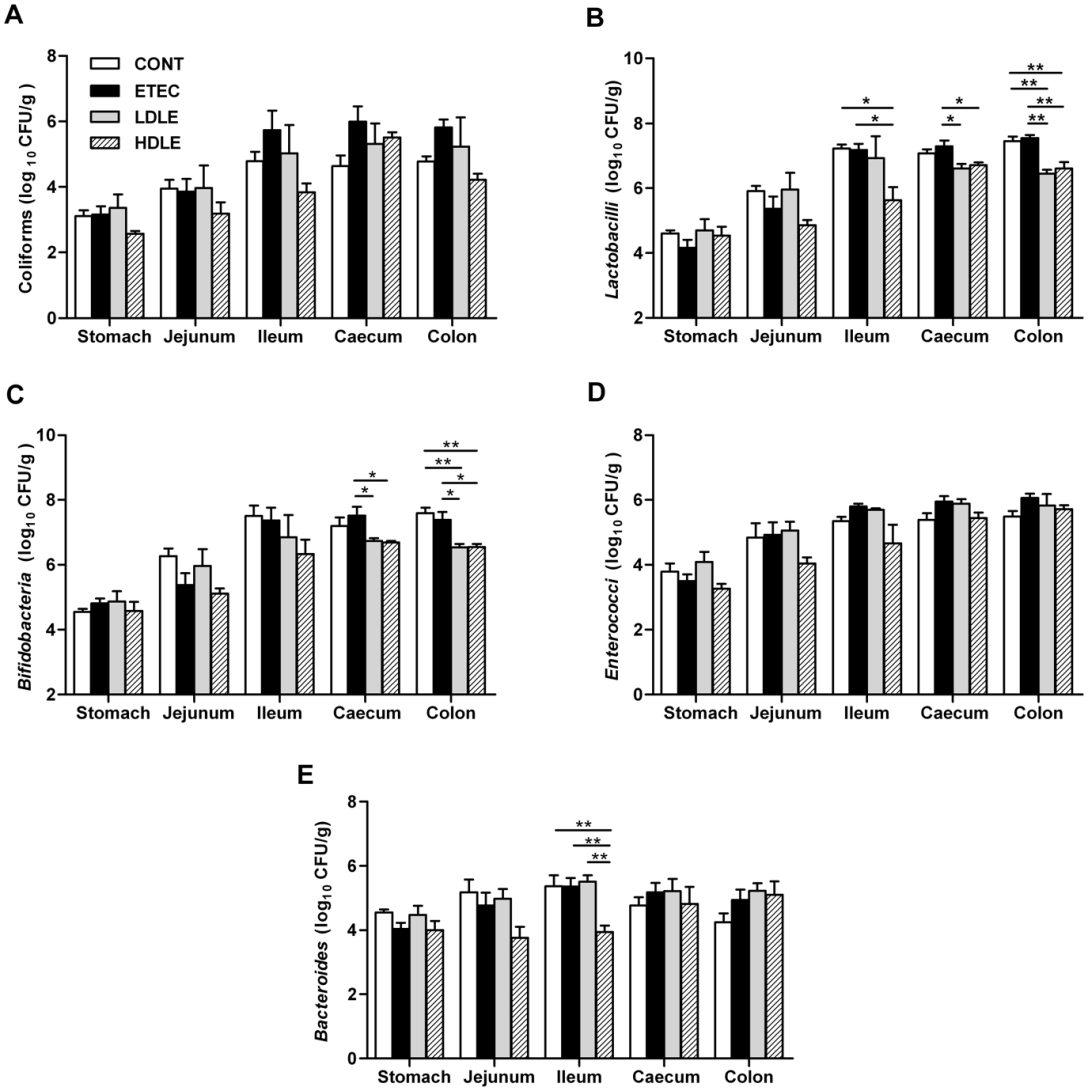
*Lactobacillus rhamnosus* consumption shaped the composition of intestinal microbiota during F4^+^ETEC infection. (A) Coliforms, (B) *Lactobacilli*, (C) *Bifidobateria*, (D) *Enterococci* and (E) *Bacteroides*. The contents from the stomach, mid-jejunum, distal ileum, caecum and mid-colon were collected at day 15 after weaning (1 week after F4^+^ETEC challenge) for microbial cultures. Results are presented as log_10_ CFU/g contents, and all counts were performed in triplicate. Data are presented as means ± SEM (n = 5 per group). *P<0.05; **P<0.01.

To test whether pretreatment with *L. rhamnosus* was able to balance the microbial ecosystem when encountering F4^+^ETEC, the microorganisms and pH values in the contents of the stomach, jejunum, ileum, caecum and colon were measured immediately after animals were sacrificed (at day 15). Unlike shedding of faecal microbes, pigs in LDLE or HDLE had lower *Lactobacillus* and *Bifidobacterium* counts in the colonic contents than did those in CONT or ETEC. Similarly, *Lactobacillus* and *Bifidobacterium* counts in the caecum were lower in LDLE or HDLE than in ETEC (Figures 3B and 3C). There were no significant differences in the number of coliforms, *Enterococci* or *Bacteroides* in the contents of the large intestine among the different groups (Figures 3A, 3D and 3E). Notably, *L. rhamnosus* administration with or without F4^+^ETEC challenge had no effect on the populations of tested microorganisms in the contents of the stomach and jejunum. In the ileal contents, pigs in HDLE but not in LDLE had lower *Lactobacillus* and *Bacteroides* counts than did those in CONT or ETEC. The pH values of the contents of the stomach, jejunum, ileum, caecum and colon did not differ among the four groups at day 15 (data not shown).

### The F4^+^ETEC-induced Increase in the Concentration of Serum TNF-α was Delayed by *L. rhamnosus* Consumption

**Figure 4:**
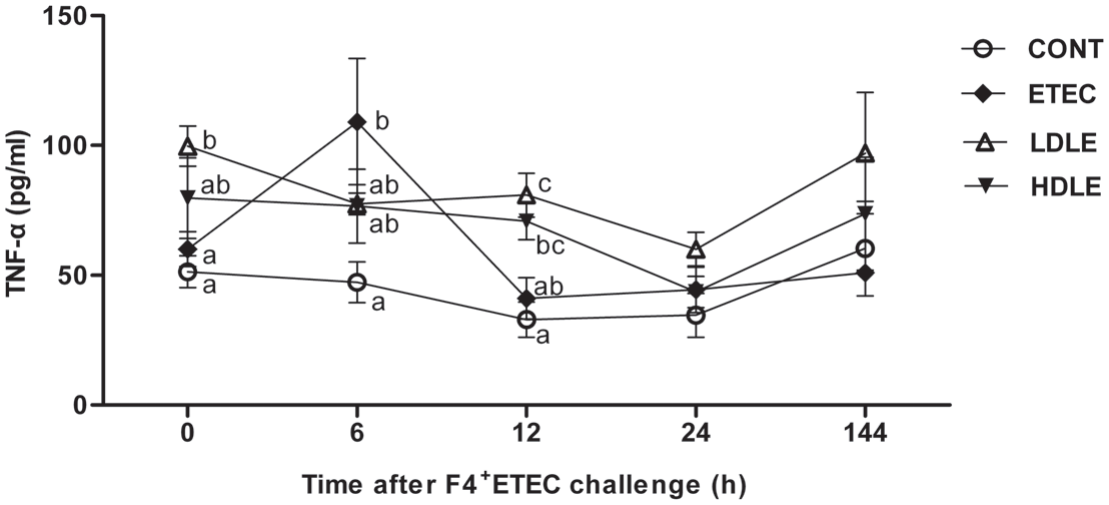
The F4^+^ETEC-induced increase in the concentration of serum TNF-α was delayed by *L. rhamnosus* consumption. Serum TNF-α concentrations were measured by ELISA at 0, 6, 12, 24 and 144 h after F4^+^ETEC challenge. Data are presented as means ± SEM (n = 5 per group). Within the same time, mean values with different superscript letters (^a, b, c^) are significantly different (P<0.05).

Tumour necrosis factor-α (TNF-α) serves as a marker of inflammation. To test whether *L. rhamnosus* can attenuate host systemic inflammatory responses induced by F4^+^ETEC, serum concentrations of TNF-α were measured immediately after F4^+^ETEC challenge (Figure 4). At 0 h before F4^+^ETEC challenge, the TNF-α concentration was higher in LDLE pigs (99.7±7.8 vs. 51.3±6.1 pg/ml, *P* = 0.035) than in CONT pigs. At 6 h after F4^+^ETEC challenge, a marked elevation (109.1±24.3 vs. 47.3±7.8 pg/ml, *P*<0.001) of TNF-α concentration was observed in ETEC pigs compared to CONT pigs, whereas pigs pretreated with *L. rhamnosus* had attenuated F4^+^ETEC-induced acute inflammatory responses with only a moderate increase in serum TNF-α concentrations in either LDLE or HDLE pigs (77.5±4.2 or 76.7±14.2 pg/ml). At 12 h after F4^+^ETEC challenge, TNF-α concentrations in ETEC pigs decreased to a level similar to CONT pigs, whereas the concentrations of TNF-α remained higher (80.9±8.4 or 70.8±7.1 vs. 32.9±6.7 pg/ml, *P* = 0.002 or *P* = 0.008, respectively) in LDLE and HDLE pigs compared to CONT pigs. Our results indicate that the concentration of serum TNF-α increases during F4^+^ETEC infection, and that administration of *L. rhamnosus* delays this increase.

### Pretreatment with Low- or High-dose *L. rhamnosus* had Distinct Immunomodulatory Effects on the Small Intestine After Exposure to F4^+^ETEC

**Figure 5:**
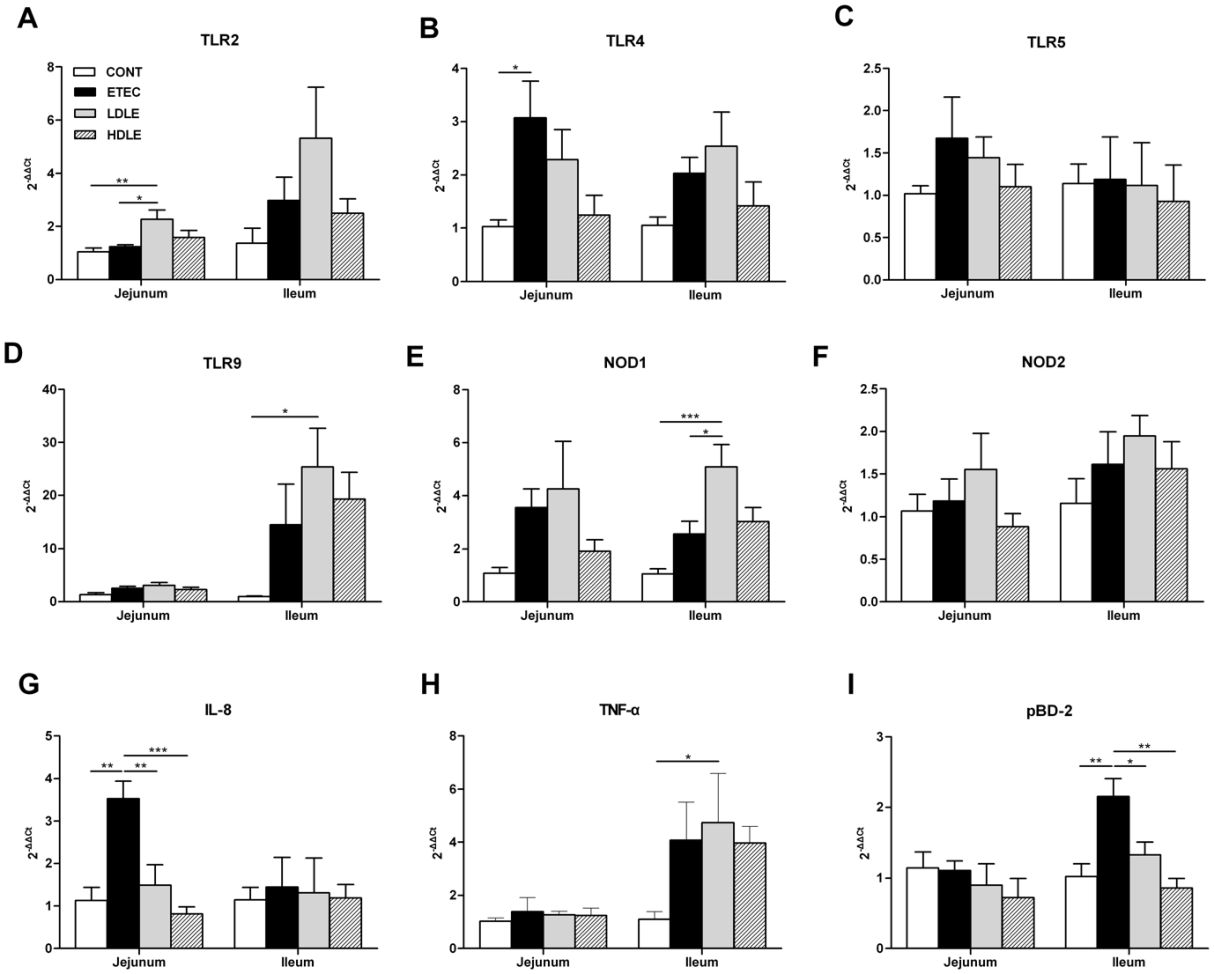
Low- or high-dose *L. rhamnosus* had distinct immunomodulatory effects on the intestines after F4^+^ETEC challenge. The expression of mRNA for genes encoding (A-D) TLR2, 4, 5 and 9, (E, F) NOD1 and NOD2, (G) IL-8, (H) TNF-a and (I) pBD-2 in both jejunum and ileum tissues collected at day 15 after weaning (1 week after F4^+^ETEC challenge) was analysed by quantitative real-time PCR. Data are presented as means ± SEM (n = 5 per group). *P<0.05; ** P<0.01; *** P<0.001.

To understand the interaction between porcine intestinal mucosa immunity and the dose effects of *L. rhamnosus* in preventing enteric infections, we quantified the expression of mRNA for selected genes encoding TLRs, NODs, pro-inflammatory cytokines and chemokines as well as antimicrobial peptides in both jejunal and ileal tissues (Figure 5).

TLRs and NODs are thought to be important in the communication between the gut and the encountered microbes, as both of these receptors play a key role in the genesis and modulation of the inflammatory response. Among TLRs, TLR2, 4, 5 and 9 are involved in detecting bacterial ligands. As expected, the expression of TLR4 mRNA in the jejunum increased significantly (*P* = 0.021) in ETEC pigs compared to CONT pigs, whereas the effect was not reproduced by pigs pretreated with *L. rhamnosus* following F4^+^ETEC challenge (Figure 5B). Conversely, a marked increase in jejunal TLR2 (*P* = 0.008) and ileal TLR9 (*P* = 0.015) mRNA expression was detected in LDLE pigs compared to CONT pigs, and the expression of jejunal TLR2 mRNA was higher (*P* = 0.016) in LDLE pigs than in ETEC pigs (Figure 5A and Figure 5D). By comparison, both TLR2 and TLR9 mRNA expressions were not differentially regulated in pigs pretreated with high-dose *L. rhamnosus.* No changes in TLR5 mRNA expression were observed in any of the intestinal tissues analysed (Figure 5C).

After F4^+^ETEC challenge, NOD1 mRNA expression increased (*P*<0.001) in ileal tissues from pigs pretreated with low- but not with high-dose *L. rhamnosus* compared to those from CONT pigs, and the expression of NOD1 mRNA was also higher (*P* = 0.019) in LDLE pigs than in ETEC pigs (Figure 5E). There were no significant differences in NOD2 mRNA expression in the intestinal tissues among the four treatment groups (Figure 5F).

We next compared the expression profile of a generalized or preparative immune/inflammatory response 1 week after F4^+^ETEC challenge in pigs pretreated with low- and high-dose *L. rhamnosus*. Although increased serum TNF-α concentrations were found during early stages of F4^+^ETEC infection (6 h after challenge), no significant differences in jejunal TNF-α mRNA expression were observed in ETEC pigs compared to CONT pigs (7 days after challenge) (Figure 5H). Instead, the expression of TNF-α was upregulated (*P* = 0.043) in the ileal tissues from pigs pretreated with low- but not with high-dose *L. rhamnosus* compared to those from CONT pigs.

Interleukin (IL)-8 acts as a strong neutrophil chemotactic factor. Exposure of pigs to F4^+^ETEC without *L. rhamnosus* administration resulted in a significant increase (*P*<0.001) in the expression of IL-8 mRNA in jejunal tissues compared to the unchallenged control, whereas IL-8 mRNA expression in pigs pretreated with *L. rhamnosus* (group LDLE or HDLE) was lower (*P* = 0.005 and *P*<0.001, respectively) than in pigs challenged with F4^+^ETEC only (Figure 5G).

Porcine β-defensins 2 (pBD-2) has broad antibiotic spectrum against pathogenic microbes. Similar to IL-8, the expression of pBD-2 mRNA in ileal tissue was markedly upregulated (*P* = 0.003, *P* = 0.033 and *P* = 0.001, respectively) in ETEC pigs compared to CONT, LDLH or HDLH pigs (Figure 5I).

### F4^+^ETEC Infection Increased TLR4 Protein Expression in the Jejunal Mucosa

**Figure 6:**
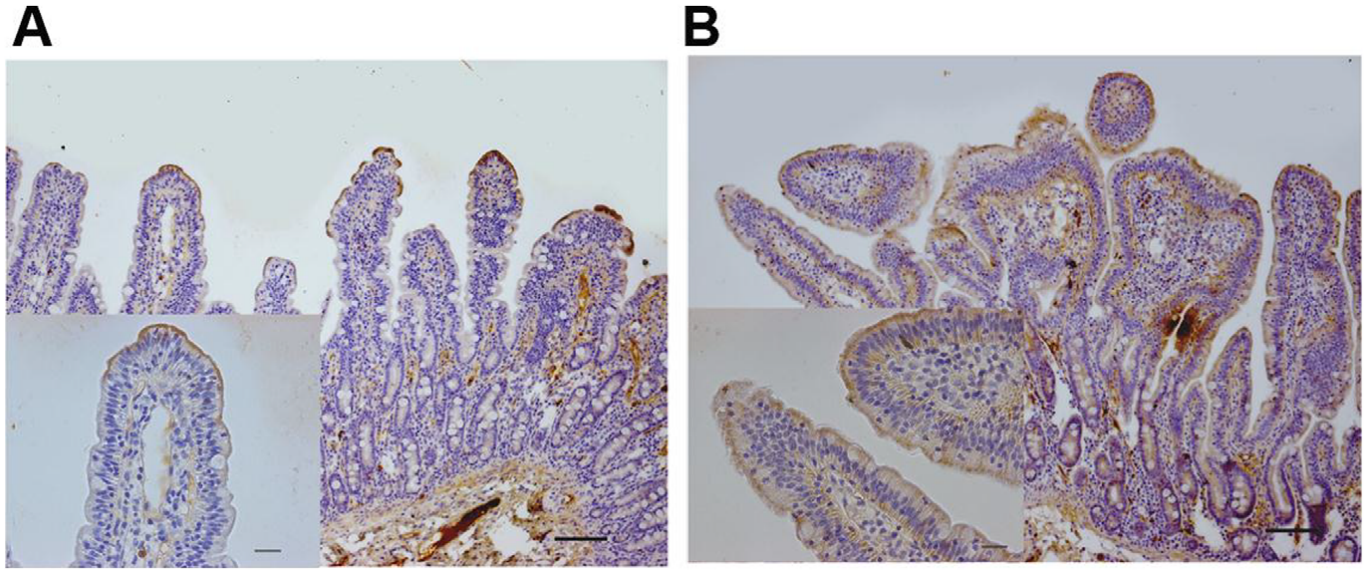
Immunohistochemical localization of TLR4 in jejunal tissues collected from piglets with or without F4^+^ETEC challenge. (A) Representative microphotographs of DAB-stained jejunum sections from one piglet that received sterile physiological saline orally at day 15 after weaning. TLR4 was localized in the superficial epithelium limited to the tips of the villi and in the mesenchymal cells within the lamina propria, with scattered positive staining. (B) Representative microphotographs of DAB-stained jejunum sections from one infected piglet at day 7 after F4^+^ETEC challenge (at day 15 after weaning). Increased TLR4 expression was observed in the epithelial cells of both the villi and the crypts and the mesenchymal cells of the lamina propria. Scale bars, 100 µm, 25 µm.

Since jejunal rather than ileal TLR4 mRNA expression was increased significantly in ETEC compared to CONT, we next sought to detect localization of TLR4 in the jejunal mucosa of pigs from both CONT and ETEC. Immunohistochemistry analysis of jejunal sections in CONT pigs revealed that TLR4 was primarily localized in the superficial epithelium limited to the tips of the villi and in the mesenchymal cells of the lamina propria, with scattered positive staining (Figure 6A), whereas ETEC pigs had increased apical cytoplasmic TLR4 protein expression in the epithelial cells of the villi and the crypts, and mesenchymal cells of the lamina propria (Figure 6B).

## Discussion

Although the role of probiotics as prophylactic or therapeutic agents in diarrhoea has been extensively studied, less information is available regarding the optimum dosing regimens for probiotics. A commonly held assumption is that higher doses of probiotics given for short courses are more effective than lower doses at ameliorating acute infectious diarrhoea; however, the dose effects of probiotics on ameliorating acute infectious diarrhoea remain controversial [25], [26]. Currently, there are few data concerning the effects of probiotics on piglet health. Weanling piglets are considered to be an ideal monogastric model for studying human gastrointestinal diseases such as diarrhoea caused by ETEC and for evaluating the efficacy of various probiotic strains [27]. Accordingly, the dose effects of probiotics on preventing F4^+^ETEC-induced diarrhoea in weanling pigs is currently being explored.

It has been proposed that probiotics modulate and stabilize the gut microbiota through competitive exclusion and enhance colonization resistance against enteric pathogens. Consistent with the results of previous studies [8], [28], the present results showed that pretreatment of piglets with *L. rhamnosus* (both low- and high-dose) ameliorates F4^+^ETEC-induced diarrhoea as indicated by increases in the number of *Lactobacilli* and *Bifidobacteria* accompanied by a reduction in coliform shedding in faeces. Indeed, an *in vitro* study found that *E. coli* adhesion to porcine intestinal mucosa could be inhibited by LGG [29]. The stress of weaning is often associated with a decreased number of *Lactobacilli* combined with an increased number of *E. coli* in the gastrointestinal tract [30]. Such shifts in the host microbial ecosystem facilitate pathogen overgrowth and subsequently trigger the occurrence of infectious diseases [31]. Treatment with specific *Lactobacillus* strains may restore the homeostasis of an impaired microbial ecosystem and confer resistance to ETEC infection in weanling piglets.

Unexpectedly, the number of *Lactobacilli* and *Bifidobacteria* in the colonic contents was decreased at day 15 in piglets pretreated with either low- or high-dose *L. rhamnosus*. Moreover, decreases in the number of *Lactobacilli* and *Bacteroides* were also observed in the ileal contents of piglets pretreated with a high dose of *L. rhamnosus*; however, the mechanism behind this phenomenon is unknown. Our findings indicate that increasing the expression of jejunal TLR2 with a stimulation of commensal bacteria may in turn increase mucosal sensitivity to commensal gram-positive bacteria, such as *Lactobacilli* and *Bifidobacteria*. Alternatively, supplementation of exogenous commensal strains may lead to a decrease in the number of closely related autochthonous strains, due in part to immune cross-reactivity and competition for limited niches or nutrition. As shown in a previous study [32], strain*-*specific recognition of different *Lactobacillus* species is partially related to immune cross-reactivity, which leads to a reduction in the total *Lactobacillus* count in the intestinal contents. It is interesting to note that probiotic consumption also affected Gram-negative *Bacteroides*. In addition to competitive exclusion or immunomodulation of host defenses, other yet-unknown bacterial properties may also be involved in the reduction in the number of intestinal microorganisms following *L. rhamnosus* consumption. Further studies are needed to specifically confirm the presence of the probiotic strain *L. rhamnosus* in faeces or gut contents and to elucidate the underlying mechanisms, particularly when different doses are used.

Notably, high-dose *L. rhamnosus* (10^12^ CFU/d) administration resulted in an increased incidence of diarrhoea before F4^+^ETEC challenge. Similarly, a recent trial of piglets challenged with F4^+^ETEC showed that dietary addition of LGG (10^10^ CFU/d) fail to prevent F4^+^ETEC infection, possibly due to the greater degree of stress caused by the challenge [9]. In fact, it has been reported that high-dose (10^10^ CFU/L) but not low-dose LGG has pro-inflammatory properties in Caco-2 cells by markedly increasing IL-8 production, which helps to understand side effects of high-dose LGG [33]. An imbalance between potentially pathogenic commensal bacteria and potentially beneficial commensal bacteria in the microbiota might play a role in pathogenesis [34]. It could thus be hypothesized that excessive probiotic challenge may disturb the balance of gastrointestinal microbial ecosystem and cause inflammatory responses, thus leading to the development of diarrhoea.

In this study, jejunal TLR4 expression at the mRNA and protein levels, and jejunal IL-8 mRNA expression were significantly elevated in piglets exposed to F4^+^ETEC. Moreover, increased intestinal TLR4 and IL-8 mRNA expression was attenuated by pretreatment with *L. rhamnosus*. Similarly, previous *in vitro* studies have shown that LGG or *L. jensenii* suppress *E. coli*-induced chemokine IL-8 expression by modulating negative signalling regulators [16], [35]. Furthermore, live *L. casei* counteracts the pro-inflammatory effects of *E. coli* on mucosa inflamed by Crohn’s disease via downregulation of IL-8 [36]. Our findings indicate that *L. rhamnosus* attenuates F4^+^ETEC-induced IL-8 expression in the intestine.

The intestinal microbiota promotes host health in part via the activation of host TLRs [37]. Among which, TLR2 recognizes lipoteichoic acid (LTA), lipoproteins, and peptidoglycans (PGN) from Gram-positive bacteria by forming a heterodimer with either TLR1 or TLR6, and TLR9 is a receptor for CpG DNA in bacterial genomes [11], [12]. Interestingly, in low- but not in high-dose *L. rhamnosus* pretreated animals, jejunal TLR2, ileal TLR9 and NOD1 mRNA expression increased after F4^+^ETEC challenge. Our findings were consistent with other studies showing that certain *Lactobacillus* strains may exert anti-inflammatory effects via TLR2 or TLR9 activation [17], [38]. More recently, an *in vitro* study using mouse enterocytes found that LPS-mediated TLR4 signalling could be inhibited by activation of TLR9 with bacterial DNA via the inhibitory kinase IRAK-M [39]. Further studies are needed to clarify the mechanisms of the effectors triggered by TLRs involved in regulating the anti-inflammatory response.

Nod-like receptors are the cytoplasmic counterparts of TLRs, which in combination with TLRs constitute a cellular defense both at the plasma membrane and within the cell [40]. Nod-like receptor NOD1 in Caco-2 cells could prevent IκB kinase and NF-κB activation in response to enteroinvasive *E. coli* infection, whereas NOD2 is likely not important in signalling the onset of NF-κB activation [41]. In spite of NOD ligands themselves being poor triggers for cytokine responses, there is evidence that NOD1 and NOD2 act in synergy with TLRs to induce maximal innate immune responses to invading bacteria [42], [43]. The present study showed that the induction of ileal NOD1 is accompanied by upregulation of TLR2 and TLR9 expression in the pigs pretreated with low-dose *L. rhamnosus*, suggesting that the anti-inflammatory effect of *L. rhamnosus* may be a result of synergistic responses of TLR2, TLR9 and NOD1.

TNF-α has remarkable functional duality and is strongly engaged both in tissue regeneration and destruction. During the inflammatory process, TNF-α is a vital pro-inflammatory mediator. Nevertheless, TNF-α is also protective of various cell types, in particular for the epithelium [44]–[46]. We showed that production of TNF-α increases during F4^+^ETEC infection, and that administration of *L. rhamnosus* delays this increase in TNF-α production. In addition, we observed an increase in ileal TNF-α mRNA expression at day 7 after F4^+^ETEC challenge in pigs pretreated with a low-dose *L. rhamnosus*. Consistent with the results of our previous study, the present data suggest that *L. rhamnosus*-induced TNF-α production may enhance innate immune defense against F4^+^ETEC [8]. Furthermore, the delayed increase in the production of F4^+^ETEC-induced TNF-α following *L. rhamnosus* administration may slow the progression of the F4^+^ETEC infection. A previous study involving mouse splenic mononuclear cells has shown that LTAs from *Lactobacilli* elicit pro-inflammatory activities by inducing TNF-α secretion through TLR2 signalling [47]. Taken together with the results presented here, we speculate that the regulation of TNF-α is perhaps closely associated with TLR2, TLR9 or NOD1 activation. Besides, the stimulation of other anti-inflammatory pathways, including the production of IL-10 and TGF-β may also be involved.

Defensins are key effector molecules of innate immunity that protect the host from infectious microbes and shape the composition of microbiota at mucosal surfaces [48], whereas high levels of defensins would prompt the host to develop an overwhelming inflammatory response, leading to complications [49]. Human β-defensin 2 in the intestinal epithelial cells is induced by peptidoglycan or LPS, through TLR2 or TLR4 signalling specifically [50]. Porcine β-defensin 2 (pBD-2) is produced in the intestine and displays broad antimicrobial activity against pathogenic intestinal bacteria [51]. Nevertheless, the change in pBD-2 mRNA expression in the intestine after *Salmonella typhimurium* infection varies among studies. One study detected an upregulation of pBD-2 in the ileum [52], whereas another study reported no change in pBD-2 expression in the proximal part of the ileum [53]. A more recent study detected no change in ileal pBD-2 expression (24 h after challenge) in pigs challenged with F4^+^ETEC [54]. The present data indicate that piglets pretreated with *L. rhamnosus* do not have further increases in ileal pBD-2 mRNA expression at day 7 after F4^+^ETEC infection, and that the F4^+^ETEC-induced increase in ileal pBD-2 expression seems to be attenuated by pretreatment of pigs with *L. rhamnosus* during ETEC infection.

The dose-dependent action of probiotics was also reflected by the gene expression profiles in our study. We found that piglets pretreated with high-dose *L. rhamnosus* had stronger downregulation of F4^+^ETEC-induced jejunal TLR4, IL-8 and ileal pBD-2 mRNA expression compared to those pretreated with low-dose, and that piglets pretreated with high-dose *L. rhamnosus* failed to upregulate TLR2, TLR9, NOD1 and TNF-α mRNA expression. Indeed, the inhibition of ETEC adherence to Caco-2 cells by *Lactobacillus* strains is dose-dependent [55], [56]. The mechanism of ETEC pathogenesis as it pertains to ETEC excretion, ETEC-specific IgA and TLR expression needs to be addressed in further studies. In the present study, one of the possible explanations for the discrepancy between the effects of the low- and high-dose administration might be that exposure to a larger primary *L. rhamnosus* challenge may induce hyporesponsiveness to the secondary F4^+^ETEC challenge. Previous studies have reported that increasing the primary stimulus induced a hyporesponsiveness toward a second stimulus [57]. Interestingly, one study reported that ingestion of *L. rhamnosus* (5×10^10^ CFU/d) by both healthy volunteers and patients with Crohn’s disease for 2 weeks induced T cell hyporesponsiveness and this was proposed as an explanation for the observed therapeutical effects of probiotics in clinical disease [58]. Therefore, large doses of probiotics (≥10^12^ CFU/d) may have side effects on ameliorating infectious diarrhoea.

In conclusion, our data indicate that administration of *L. rhamnosus* shapes the composition of the gut microbiota, attenuates acute inflammatory responses induced by F4^+^ETEC, and slows the progression of acute infectious diarrhoea, thus ameliorating F4^+^ETEC-induced diarrhoea in piglets. Of note, the results of our study contradict the assumption that the beneficial effects of probiotics in preventing infectious diarrhoea accumulate with increasing dose. Instead, we found that pretreatment with a low dose of *L. rhamnosus* might be more effective at ameliorating diarrhoea than pretreatment with a high dose. High doses of certain probiotics may negate the preventative effects, at least in part by disturbing the established microbial ecosystem and by interfering with mucosal immune responses against potential enteric pathogens. These data could provide an important contribution to the development of an optimal dosing regimen for preventing acute infectious diarrhoea.

## Materials and Methods

### Ethics Statement

All animals were treated in strict according to the guidelines for the Laboratory Animal Use and Care from Chinese Center for Disease Control and Prevention and the Rules for the Medical Laboratory Animal (1998) from Ministry of Health, China, under the protocol (CAU-AEC-2011-056) approved by the Animal Ethics Committee of the China Agricultural University. All surgery was performed under sodium pentobarbital anesthesia, and every effort was made to minimize suffering.

### Animals

Twenty crossbred (Landrace × Large White) piglets weaned at 21 days of age and weighing 5.2±0.2 kg were obtained from a commercial farm in Beijing. At weaning, pigs were transported to the animal experimental facility of the College of Veterinary Medicine, China Agricultural University. The animals were individually housed in wire-mesh pens, each of which was equipped with a single feeder and a nipple drinker, and fed with a standard weaner diet containing 22.3% crude protein and 14.0 MJ/kg dietary energy. Feed and water were provided *ad libitum*. None of the diets contained antibiotics and no drug was administered throughout the trial. Prior to the start of the trial, no clinical signs of diarrhoea or other diseases were observed in any of the piglets. The pigs were weighed before the start of the trial (day 0), at day 8 (pre-challenge) and at day 15 (when slaughtered). The individual feed intake of each pig was recorded.

### Bacterial Strains

*Lactobacillus rhamnosus* 1.120 ( =  ATCC 7469) purchased from the Chinese General Micro-organism Culture Collection was grown in De Man, Rogosa and Sharpe (MRS) broth (Oxoid, Hampshire, United Kingdom) for 24 h at 37°C under microaerophilic conditions and subsequently harvested by centrifugation at 2000×g for 5 min at 4°C, then washed 3 times with sterile physiological saline and resuspended in saline. Two doses of *L. rhamnosus* containing approximately either 1×10^9^ CFU/ml or 1×10^11^ CFU/ml were prepared.

*Escherichia coli* F4-producing strain (O149:K91, K88ac) obtained from the China Veterinary Culture Collection Center was grown in Luria-Bertani (LB) medium (Oxoid, Basingstoke, England). After overnight incubation at 37°C with shaking, bacteria were diluted 1∶100 in fresh LB. Following incubation, the bacterial cells were harvested by centrifugation at 3000×g for 10 min at 4°C, washed in sterile physiological saline and resuspended in saline. An inoculum of F4^+^ETEC containing approximately 1×10^9^ CFU/ml was prepared.

### Experimental Design

On the day of weaning (day 0), pigs were assigned to 4 groups (n = 5 per group), with littermates and mean initial body weights distributed evenly among the groups. The following four groups were: 1) CONT, pigs orally administered with sterile physiological saline; 2) ETEC, pigs orally administered with sterile physiological saline and orally challenged with ETEC culture; 3) LDLE, pigs orally administered with low-dose *L. rhamnosus* and orally challenged with ETEC culture; and 4) HDLE, pigs orally administered with high-dose *L. rhamnosus* and orally challenged with ETEC culture.

At 9∶00 every day for the first week (from day 1 to day 7), pigs in groups CONT and ETEC were orally administered 10 ml of sterile physiological saline, whereas pigs in groups LDLE and HDLE were orally administered an equal volume of low-dose *L. rhamnosus* solution (10^9^ CFU/ml, 10 ml/day) or high-dose *L. rhamnosus* solution (10^11^ CFU/ml, 10 ml/day), respectively. At 9∶00 on day 8, pigs in groups ETEC, LDLE and HDLE were orally administered 10 ml of ETEC culture (10^9^ CFU/ml) to induce diarrhoea as described, whereas pigs in group CONT received the same amount of sterile physiological saline.

Health status of each animal was closely monitored throughout the experiment, with special attention to faecal consistency. Severity of diarrhoea was scored according to the following criteria [59]: 1, hard and formed pellets; 2, nonformed pellets; 3, soft faeces; 4, very soft and containing a small amount of water-like faeces; 5, semisolid containing more than half water-like faeces; 6, water-like faeces. Piglets were considered to have severe diarrhoea when the score was 5 or 6. Fresh faecal samples from all animals were collected at days 1, 3, 5, 7, 9, 11 and 13 for microbial cultures.

Blood samples were collected just before F4^+^ETEC challenge (0 h) and at 6, 12, 24, and 144 h after challenge. At 9∶00 on day 15 (1 week after F4^+^ETEC challenge), intestinal samples were collected immediately after euthanasia. For mRNA studies, middle jejunum and ileum segments collected from each pig were immediately frozen in liquid nitrogen and stored at −80°C. For immunostaining studies, proximal jejunum segments were rinsed immediately after being opened, segmentally divided and immersed in 4% paraformaldehyde. The contents from the stomach, mid-jejunum, distal ileum, caecum and mid-colon were sampled for pH measurement and microbial cultures.

### Bacterial Enumeration

Each fresh faecal sample or gastrointestinal content (1 g) was homogenized in 9 ml sterile saline solution, and suitable dilutions of the homogenates were then plated onto selective medium. The groups of faecal and gastrointestinal microbes studied and the selective mediums (Beijing Land Bridge Technology Co., China) employed were as follows: 1) Coliforms, Eosin-Methylene Blue (EMB) agar; 2) *Lactobacilli*, MRS agar; 3) *Bifidobateria*, Tryptonephytone Yeast (TPY) agar; 4) *Enterococci*, Pfizer agar; 5) *Bacteroides*, Biotin vitamers D-biotin-d-sulfoxide (BDS) Medium. The plates for EMB agar and Pfizer agar were incubated for 24 h at 37°C under aerobic conditions, whereas MRS agar, TPY agar and BDS plates were incubated under anaerobic conditions for 48 h at 37°C. Results were presented as log_10_ CFU/g faeces or contents, and all counts were performed in triplicate.

### ELISA

Serum TNF-αconcentrations were determined by means of commercially available porcine TNF-α ELISA kit (R&D Systems, Minneapolis, MN). The dynamic range of the TNF-α assay was 23.4 to 1500 pg/ml. The minimum detectable dose was 3.7 pg/ml. The intra-assay and the inter-assay coefficient of variations were <4.2% and <6.5%, respectively.

**Table 1:** Sequences of oligonucleotide primers used for real-time PCR, length of the PCR products and the accession numbers.

Gene	Primer sequences (5′to3′)		Product size (bp)	Accession number	Ref.
HRRT	F	GTGATAGATCCATTCCTATGACTGTAGA	104	U69731	[60]
	R	TGAGAGATCATCTCCACCAATTACTT			
TLR2	F	ACATGAAGATGATGTGGGCC	109	AB072190	[61]
	R	TAGGAGTCCTGCTCACTGTA			
TLR4	F	GCCATCGCTGCTAACATCATC	108	NM_001113039	[61]
	R	CTCATACTCAAAGATACACCATCGG			
TLR5	F	CAGCGACCAAAACAGATTGA	122	NM_001123202	[52]
	R	TGCTCACCAGACAGACAACC			
TLR9	F	GTGGAACTGTTTTGGCATC	199	NM_213958.1	[62]
	R	CACAGCACTCTGAGCTTTGT			
NOD1	F	ACCGATCCAGTGAGCAGATA	140	NM_001114277	[52]
	R	AAGTCCACCAGCTCCATGAT			
NOD2	F	GAGCGCATCCTCTTAACTTTCG	66	NM_001105295	[20]
	R	ACGCTCGTGATCCGTGAAC			
IL-8	F	TCCTGCTTTCTGCAGCTCTC	176	NM_213867	[63]
	R	GGGTGGAAAGGTGTGGAATG			
TNF-α	F	GCCCACGTTGTAGCCAATGTCAAA	99	NM_214022	[64]
	R	GTTGTCTTTCAGCTTCACGCCGTT			
pBD-2	F	CTGTCTGCCTCCTCTCTTCC	148	NM_214022	[52]
	R	CAGGTCCCTTCAATCCTGTT			

HPRT =  hypoxanthine phosphoribosyl-transferase; TLR  =  Toll-like receptor; NOD  =  nucleotide binding oligomerization domain; IL  =  interleukin; TNF  =  tumor necrosis factor; pBD-2 =  porcine β-defensin-2; F  =  forward primer; R  =  reverse primer; Ref.  =  reference.

### Isolation of Total RNA and Reverse Transcription

Total RNA was extracted from the jejunal and ileal tissue samples using Trizol reagent (Invitrogen, Carlsbad, CA). The final RNA was eluted in an appropriate amount of 0.1% Diethyl pyrocarbonate (DEPC) treated water (Sigma-Aldrich, Inc., Saint Louis, MO). For each sample, the integrity of RNA extracted was confirmed by agarose gel electrophoresis by staining with ethidium bromide and visualization under UV light. The amount of RNA extracted was determined and its purity (OD260/OD280 absorption ratio >1.9) was verified using a NanoDrop® ND-2000C Spectrophotometer (NanoDrop Technologies Inc., Wilmington, DE). For cDNA synthesis, a 2 µg aliquot of total RNA was reverse-transcribed with 200 U M-MLV (Promega, Madison, WI) using 1 µg oligo (dT)_15_ Primer, 10 mM dNTP Mix, M-MLV 5x Reaction Buffer, 25 U rRNasin® Ribonuclease Inhibitor (Promega, Madison, WI) in a final volume of 25 µl. To inspect DNA contamination, a negative control (without enzyme) was included. Synthesized cDNA was stored at −20°C prior to real-time PCR analysis.

### Quantitative Real-time PCR

Quantitative real-time PCR was performed using an ABI 7500 Real-time PCR System (Applied Biosystems, Foster City, CA). The sequences of primers used were listed in Table 1. Primers for specific porcine genes were synthesized (TakaRa Biotechnology Inc., Dalian, China) to have an equal annealing temperature of 60°C. The cDNA was amplified with SYBR® Premix DimerEraser™ (TakaRa Biotechnology Inc., Shiga, Japan) containing 2 µl cDNA, 1.0 µM primers, 10 µl 2x SYBR Premix DimerEraser, 0.4 µl ROX (passive reference dye).The PCR amplification was performed using the following conditions: 95°C for 30 s, followed by 39 cycles at 95°C for 5 s, 60°C for 30 s and 72°C for 15 s. Melting curve analysis was systematically analysed after each run. All melt curves showed a single peak and were consistent with the presence of a single amplicon. A non-template control of nuclease-free water was included in each run. All reactions were conducted in triplicate.

To evaluate the relative quantification of mRNA expression, the cycle threshold (C_T_) values of the target genes were normalized to the C_T_-values of the housekeeping gene hypoxanthine phosphoribosyl-transferase (HPRT) and the results were presented as fold changes using the 2^−ΔΔCT^ method. The relative mRNA expression of the target genes in each group was calculated using the following equations: ΔC_T_  =  C_T(target gene)_ – C_T(HRRT)_, ΔΔC_T_  =  ΔC_T (treated group)_ – ΔC_T (control group)._

### Immunhistochemistry

The jejunal tissue samples fixed in 4% paraformaldehyde were embedded in paraffin and sectioned into 3 µm, then collected on silanized slides. After deparaffinization and hydration through xylenes, slides were subjected to microwave for antigen retrieval and then cooled down at room temperature. Endogenous peroxidase activity was quenched with 3% H_2_O_2_ in methanol for 20 min at room temperature, followed by incubation with rabbit serum of the species from which the secondary antibody was produced for 30 min. Subsequently, sections were incubated overnight at 4°C in a humidified chamber with a 1∶100 dilution of the primary antibody, goat anti-human TLR4 polyclonal antibodies (R&D Systems Inc., Minneapolis, MN).

TLR4 was then detected using a commercial immunoperoxidase staining kit (Vectastain Elite ABC Kit; Vector Laboratories, Burlingame, CA). Briefly, the sections were incubated with a 1∶200 dilution of biotinylated secondary rabbit anti-goat antibody for 2 h at room temperature, followed by the avidin-biotin-peroxidase complex (ABC) reagent incubation for 1 h. Bound antibody conjugates were visualized using 3, 3′-diaminobenzidine (DAB) (Zhongshan Golden Bridge Biotechnology Co., Beijing, China) as a chromogen to develop a brown staining and sections were counterstained with hematoxylin and mounted with glycerol gelatin. Negative controls were included in each batch and performed with the same procedure by replacing the primary antibody with PBS. The slides were visualized and photographed using a Nikon Eclipse T*i*-U inverted microscope equipped with a Nikon DS cooled camera head (Nikon, Tokyo, Japan).

### Statistical Analysis

Statistical evaluation was performed using the SAS statistical software package, version 9.1 (SAS Institute, Inc., Cary, NC). Data were analysed using the SAS software PROC MIXED procedures. The statistical model included the effect of treatment, litter, sampling time or different intestinal sections, and the random effect of pig within treatment. When an overall effect of treatment was observed (p<0.05), differences between the least square means were compared using Tukey’s test. A value of p<0.05 was considered statistically significant.
